# Differential expression of anterior gradient gene AGR2 in prostate cancer

**DOI:** 10.1186/1471-2407-10-680

**Published:** 2010-12-13

**Authors:** Erin L Maresh, Vei Mah, Mohammad Alavi, Steve Horvath, Lora Bagryanova, Emily S Liebeskind, Laura A Knutzen, Yong Zhou, David Chia, Alvin Y Liu, Lee Goodglick

**Affiliations:** 1 Department of Pathology and Laboratory Medicine, David Geffen School of Medicine, University of California, Los Angeles, 10833 Le Conte Ave., Los Angeles, CA 90095, USA; 2 Department of Biostatistics, David Geffen School of Medicine, University of California, Los Angeles, 10833 Le Conte Ave., Los Angeles, CA 90095, USA; 3 Department of Human Genetics, David Geffen School of Medicine, University of California, Los Angeles, 10833 Le Conte Ave., Los Angeles, CA 90095, USA; 4 Department of Urology, University of Washington, 1959 NE Pacific St., Seattle, WA 98195, USA; 5 Institute for Stem Cell and Regenerative Medicine, University of Washington, 1959 NE Pacific St., Seattle, WA 98195, USA; 6 Institute for Systems Biology, University of Washington, 1959 NE Pacific St., Seattle, WA 98195, USA; 7 Jonsson Comprehensive Cancer Center, David Geffen School of Medicine, University of California, Los Angeles, 10833 Le Conte Ave., Los Angeles, CA 90095, USA

## Abstract

**Background:**

The protein AGR2 is a putative member of the protein disulfide isomerase family and was first identified as a homolog of the *Xenopus laevis *gene XAG-2. AGR2 has been implicated in a number of human cancers. In particular, AGR2 has previously been found to be one of several genes that encode secreted proteins showing increased expression in prostate cancer cells compared to normal prostatic epithelium.

**Methods:**

Gene expression levels of AGR2 were examined in prostate cancer cells by microarray analysis. We further examined the relationship of AGR2 protein expression to histopathology and prostate cancer outcome on a population basis using tissue microarray technology.

**Results:**

At the RNA and protein level, there was an increase in AGR2 expression in adenocarcinoma of the prostate compared to morphologically normal prostatic glandular epithelium. Using a tissue microarray, this enhanced AGR2 expression was seen as early as premalignant PIN lesions. Interestingly, within adenocarcinoma samples, there was a slight trend toward lower levels of AGR2 with increasing Gleason score. Consistent with this, relatively lower levels of AGR2 were highly predictive of disease recurrence in patients who had originally presented with high-stage primary prostate cancer (P = 0.009).

**Conclusions:**

We have shown for the first time that despite an increase in AGR2 expression in prostate cancer compared to non-malignant cells, relatively lower levels of AGR2 are highly predictive of disease recurrence following radical prostatectomy.

## Background

It is estimated that in 2010, there will be over 217,000 new cases of prostate cancer and over 32,000 deaths from this disease in the United States alone, making it the most common male cancer and the second leading cause of cancer-related deaths in men [1]. Prostate cancer is generally diagnosed by serum level of prostate-specific antigen (PSA) and digital rectal exam. However, although PSA is abundantly synthesized by the prostate, it is not cancer specific, leading to many unnecessary biopsies. Improved markers for both identifying prostate cancer and predicting its outcome are needed. Our approach to discover such markers involved comparative analysis of the transcriptomes of cancer cells and normal cells. These transcriptomes were determined through sorting of specific cell types from appropriate tissue specimens for analysis by Affymetrix DNA microarrays [2]. Genes that were found overexpressed by 8-fold or more in cancer cells were biomarker candidates, especially those that encode secreted or extracellular proteins. The coding sequences were analyzed with SignalP 2.0 [3] for signal peptides and TMHMM [4] for protein topology and the number of transmembrane helices. AGR2 (anterior gradient 2) was one among several such genes identified as overexpressed in prostate cancer cells. The array signal level for AGR2 in the cancer cells was ~50-fold higher than that in luminal cells, the normal counterpart [5].

AGR2 is the human homolog of the protein XAG-2 in *Xenopus laevis *and was first identified as differentially expressed in estrogen receptor positive breast cancer cell lines [6]. Biochemically, AGR2 is classified as a member of the protein disulfide isomerase (PDI) family based on amino acid sequence homology [7]. PDI enzymes in the endoplasmic reticulum act as molecular chaperones for protein folding. As such, AGR2 is thought to act as a chaperone to clear misfolded proteins out of cells during periods of physiological stress [8]. Studies have found that AGR2 expression can be increased in response to physiological stress in breast cancer cells and can enhance survival of damaged lung cancer cells [8,9]. PDI enzymes are found in other subcellular compartments and are thought to participate in or modulate a range of functions, from cell adhesion to DNA binding [10-12].

Several cancers, including breast [13-16], prostate [17-21], fibrolamellar [22], pancreatic [23-25], and colon [26], have been found to express increased levels of AGR2 compared to normal tissue. Furthermore, AGR2 has been shown to increase tumor cell migration *in vitro *and the incidence of metastatic lesions *in vivo *[15,18,21,27]. Accordingly, some studies have found AGR2 to be a marker of poor prognosis in human breast and prostate cancers [14,19,28,29]. One the other hand, some studies have found AGR2 to show no association with patient outcome in lung and pancreatic cancer [23,30] or even improved outcome in breast cancer [13].

To further evaluate the utility of AGR2 as a cancer biomarker, we examined the expression pattern of AGR2 in prostate cancer using both gene expression analysis and a high-density tissue microarray.

## Methods

### Gene expression analysis of AGR2 in prostate cancer

Two publically available datasets were used to examine AGR2 gene expression in human prostate samples [31,32]. The first dataset was generated using Affymetrix U95B Array (GEO Accession number GDS2546) [31]. It included 66 prostate cancer tissues, 17 normal prostate tissues, and 25 metastatic prostate tumor samples obtained from 4 patients. The second data set was generated using a cDNA microarray from the Stanford Microarray Database (GEO Accession number GSE3933) [32]. The dataset included 62 primary tumor tissues, 41 matched normal prostate tissues, and 9 unmatched pelvic lymph node metastases. For all datasets we used the peer-reviewed normalization procedures described by the authors. We downloaded the normalized data from the GEO database.

### Prostate tissue microarray analysis

Formalin-fixed, paraffin-embedded archival tumor specimens were obtained from 187 patients who underwent radical retropubic prostatectomy between 1984 and 1995 at the UCLA Medical Center. The construction of the prostate tissue microarray (TMA) and the utilization of the TMA were performed under appropriate UCLA IRB approval. Case material was reviewed for TMA construction by a study pathologist. At least three core tissue biopsies 0.6 mm in diameter were taken from morphologically representative regions of each prostate tumor and arrayed as previously described [33-35]. Matched benign (morphologically normal or benign prostatic hyperplasia/BPH) and *in situ *lesions of prostatic intraepithelial neoplasia (PIN) were also included. The prostate TMA was evaluated for AGR2 expression using a standard immunohistochemistry protocol as previously described [33-36]. Briefly, 4 micron sections were cut from each array block. Following deparaffinization and antigen retrieval, endogenous peroxidases were quenched and tissue was blocked with goat serum. Slides were incubated with rabbit polyclonal anti-AGR2 antibody at 1:400 (NB110-17780, Novus Biologicals, Littleton, CO) overnight at 4°C. Specific staining was detected by applying goat anti-rabbit horseradish peroxidase-conjugated secondary antibody and avidin-biotin complex followed by diaminobenzidine (Vector ABC, Burlingame, CA). If a tissue was positive for expression, only glandular epithelium stained. Moreover, the anti-AGR2 staining showed a dose-dependent titration of signal. Human colonic epithelium was used as a positive control for antibody staining. For negative control, tissue was incubated with concentration-matched non-immune rabbit IgG. Negative controls demonstrated no staining.

Staining frequency and intensity of AGR2 expression on the TMA was assessed by our pathologist (M.A.) and spot-checked by a second pathologist (V.M.) as described previously [34,36]. The correlation coefficient between scores from the two pathologists was r = 0.95. The percentage of glandular cells staining was scored from 0-100% and the intensity of staining was rated from 0 for below the level of detection to 3 for strong expression. An integrated measure of expression for frequency and intensity of staining was calculated using the following formula: [3(%x)+2(%y)+1(%z)]/100, where x, y, and z represented the percentage of cells staining at intensity 3, 2, and 1, respectively. For outcomes analysis, a mean pooled value for each case was determined as described previously [34-37].

### Statistical analysis

Statistical analyses were conducted as previously reported [34,36-38]. Briefly, non-parametric two-group and multi-group comparisons were carried out using Mann-Whitney and Kruskal-Wallis tests. Correlations were calculated using Spearman correlations. Patients were dichotomized at the 75^th ^percentile of AGR2 expression, and survival curves were visualized using the Kaplan-Meier plot with the difference between survival distributions assessed by the log-rank test. The Cox proportional hazards model was used to test the statistical significance of predictors in both a univariate and a multivariate setting. All statistical analyses were performed with StatView Version 5.0 (SAS Institute, Cary, NC) or with the freely available software package R http://www.r-project.org.

## Results

### AGR2 mRNA expression in human prostate tissue

**Figure 1:**
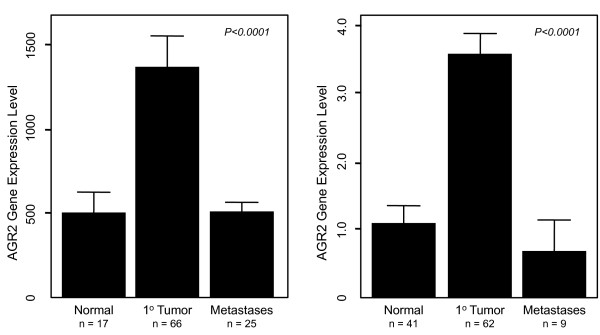
**AGR2 transcript expression in prostate cancer**. Two publically available datasets were used to examine AGR2 gene expression in human prostate samples [31,32]. (A) Dataset was from Yu, *et al*., and was generated using Affymetrix U95B Array [31]. (B) Dataset was from Lapointe, *et al*., and was generated using a cDNA microarray from the Stanford Microarray Database [32]. For each dataset, normalized data was available from the GEO database. The bars are the relative mean expression value ± standard error of the mean. P < 0.0001 for each multi group comparison (Kruskal-Wallis).

Several malignancies have been shown to express increased levels of AGR2 compared to non-malignant tissue. To further evaluate the utility of AGR2 as a cancer biomarker, we examined the gene expression pattern of AGR2 in human prostate cancer compared to non-malignant prostatic glandular epithelium. To do this we analyzed AGR2 expression from two large publically available datasets from the GEO database from which normalized results were available [31,32]. Figure 1A and 1B show mean relative expression values for each dataset. In both populations, AGR2 transcript levels were significantly higher in primary prostate cancer tissue compared to non-malignant prostate. Interestingly, transcript levels of AGR2 in metastatic lesions were also lower than the associated primary tumor (Figure 1A and 1B).

### Differential AGR2 expression in prostate cancer tissue

**Figure 2:**
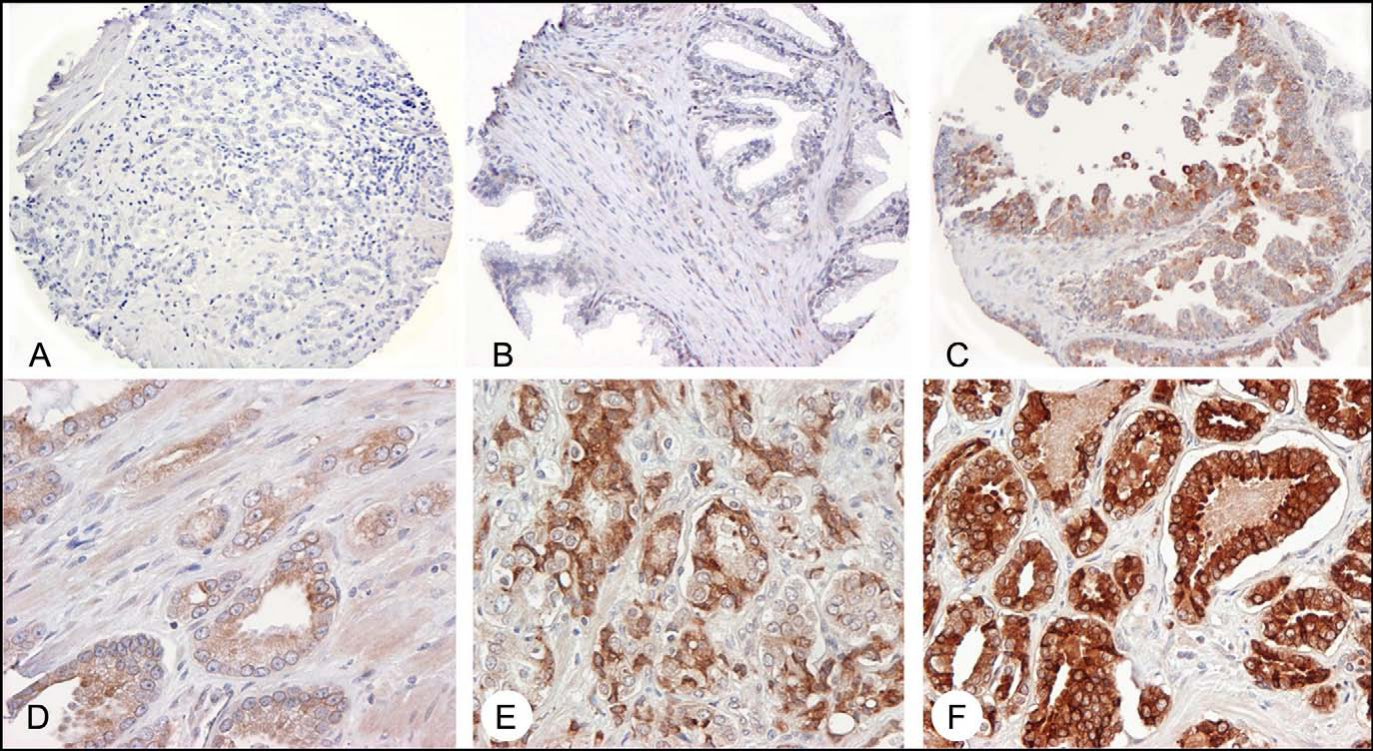
**AGR2 immunohistochemistry**. AGR2 staining was localized to the cytoplasm and membrane of cancer epithelial cells. (A) Tissue samples incubated with concentration-matched non-immune rabbit IgG showed no staining. Shown are representative sections of (B) morphologically normal tissue, (C) PIN, and (D) a representative adenocarcinoma (Gleason grade 7) showing low staining intensity, (E) a representative adenocarcinoma (Gleason grade 8) showing moderate staining intensity, and (F) a representative adenocarcinoma (Gleason grade 6) showing high staining intensity.

**Figure 3:**
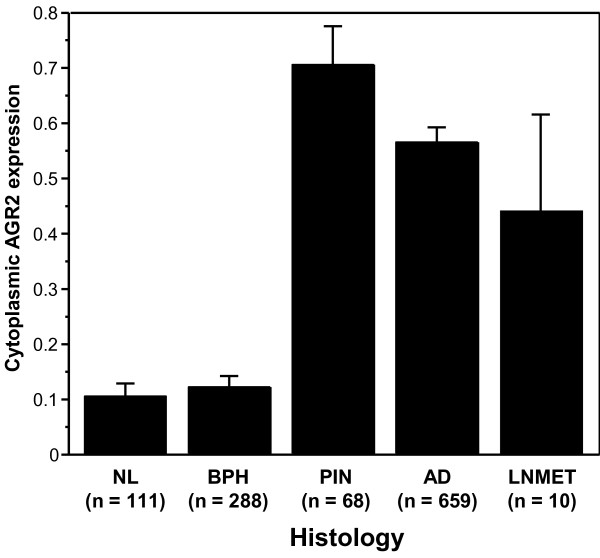
**AGR2 expression by spot level histopathology**. The barplots show the mean integrated AGR2 expression by TMA spot-level histology in morphologically normal (NL), BPH, PIN, adenocarcinoma (AD), and lymph node metastases (LNMET); bars are standard errors. Levels of AGR2 expression were increased in PIN lesions and adenocarcinoma compared to BPH and morphologically normal adjacent tissue (P < 0.0001). Regional metastases showed increased AGR2 expression compared to BPH and normal tissue (P = 0.027 and P = 0.030, respectively).

**Figure 4:**
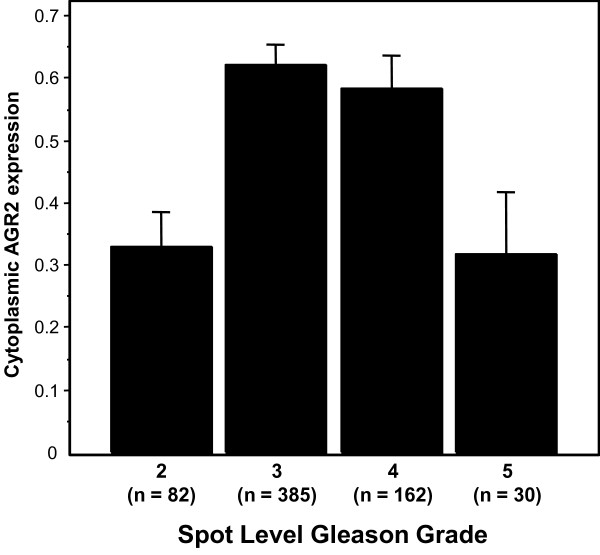
**AGR2 expression by spot level Gleason grade**. The barplots show the mean integrated AGR2 expression by TMA spot-level Gleason grade; bars are standard errors. Average AGR2 expression was increased in grades 3 and 4 compared to grade 2 (P < 0.0001 and P = 0.0007) and grade 5 (P = 0.0028 and P = 0.0085).

We further considered AGR2 protein expression in a patient cohort using a prostate TMA. The TMA consisted of specimens from 226 cases, of which 187 were informative. Cases were uninformative if their tissue spots contained no relevant epithelial cells (i.e., tumor or benign), if spots were physically missing, or if, for outcomes data, there was no information on disease recurrence. The characteristics of the patients on the TMA are listed in Table 1. AGR2, if present, was predominantly expressed in the cytoplasm of epithelial cells (Figure 2). When we examined AGR2 expression for each array spot, the level of AGR2 expression was increased in PIN lesions as well as adenocarcinoma compared to BPH and morphologically normal adjacent tissue (P < 0.0001, Figure 3). This was consistent with the cancer-specific expression of AGR2 shown in Figure 1. Representative images of weak, moderate, or strong AGR2 expression are shown in Figure 2. Overall, regional metastases tended to show AGR2 expression higher than BPH and normal tissue (P = 0.027 and P = 0.030, respectively; Figure 3). Moreover, there was an increase in AGR2 expression in Gleason grades 3 and 4 compared to 2 (P < 0.0001 and P = 0.0007; Figure 4). There was a significant drop in expression in grade 5 tumors compared to grades 3 and 4 (P = 0.0028 and P = 0.0085; Figure 4).

**Table 1:** Clinicopathologic parameters and AGR2 expression in patients with prostate cancer

	All patients	Mean AGR2 expression (SE)	P-value
Total cases	187	0.548 (0.040)	
Age at surgery			0.929^2^
Median (range)	65 (46-76)		
Mean	63.7		
Gleason score			0.986^1^
2-6	107	0.544 (0.052)	
7-10	80	0.553 (0.064)	
Stage			
I/II	122	0.559 (0.054)	0.764^1^
III/IV	65	0.527 (0.057)	
pT stage			0.767^1^
pT2-pT3a	152	0.560 (0.047)	
pT3b	35	0.495 (0.076)	
Lymph node status			0.180^1^
Positive	11	0.353 (0.118)	
Negative	174	0.564 (0.043)	
Tumor margins			0.347^1^
Positive	64	0.490 (0.060)	
Negative	123	0.578 (0.053)	
Capsular invasion			0.484^1^
No invasion	43	0.573 (0.107)	
Invasion	144	0.540 (0.042)	
Organ confined			0.477^1^
Yes	93	0.598 (0.066)	
No	94	0.498 (0.047)	
PSA ng/mL			0.339^2^
Median (range)	9.6 (0.6-96.5)		0.413^1^
Mean	14.0		
< 10	88	0.509 (0.057)	
≥10	79	0.563 (0.064)	

^1^Mann-Whitney, ^2^Spearman Correlation. Cases that were uninformative for a given variable were removed from the statistical analysis.

### Decreased AGR2 expression in high-stage prostate cancer predicts greater probability of recurrence

**Figure 5:**
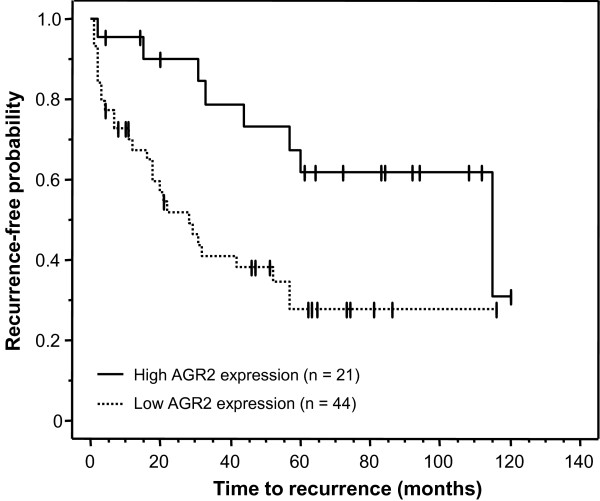
**AGR2 levels predict prostate cancer recurrence in high stage patients**. Solid line is relatively higher AGR2 levels (staining measure ≥0.79); dashed line is relatively lower AGR2 levels (< 0.79). In men with stage III or IV prostate cancer, relatively lower levels of AGR2 predict a significantly greater chance of prostate cancer recurrence compared to those with higher levels (P = 0.009).

The differential AGR2 expression in prostate cancer prompted us to examine possible links between expression and clinical outcomes. The outcomes measure we considered was tumor recurrence following radical prostatectomy as diagnosed by measurable blood PSA. AGR2 expression was not predictive of tumor recurrence either as a continuous or dichotomized variable (P = 0.596 and P = 0.281, Table 2). Patients were also grouped into high stage (III and IV) versus low stage (I and II) subpopulations. AGR2 provided no predictive value for recurrence for individuals with low stage cancer. In contrast, AGR2 was a strong predictor of tumor recurrence for individuals with high stage prostate cancer (Figure 5). Specifically, relatively lower levels of AGR2 predicted a significantly greater chance of prostate cancer recurrence compared to higher levels (P = 0.009). The median recurrence-free time in the lower AGR2 group was 14 months compared to 38.5 months in the group with relatively higher AGR2 expression.

**Table 2:** Univariate Cox model

	All Patients (n = 187)			Stage III/IV Patients (n = 65)		
Variable	HR	95% CI	P-Value	HR	95% CI	P-Value
AGR2 continuous	0.89	0.57 - 1.38	0.596	0.50	0.24 - 1.04	0.062
AGR2 dichotomized	0.75	0.44 - 1.27	0.281	0.37	0.17 - 0.81	0.013
Gleason sum^1^	3.78	2.26 - 6.33	< 0.0001	1.88	0.90 - 3.93	0.093
Preoperative PSA	1.02	1.00 - 1.03	0.011	1.00	0.98 - 1.02	0.884
pT stage^2^	3.89	2.36 - 6.40	< 0.0001	2.63	1.26 - 5.49	0.010

^1^Gleason sum was dichotomized as low (< 7) vs. high (7-10).

^2 ^pT stage was dichotomized as pT2-pT3a vs. pT3b.

Although AGR2 expression was associated with longer time to recurrence in higher stage cancers, AGR2 did not significantly associate with any other clinicopathological variables in this subgroup, such as Gleason score, lymph node status, tumor margins, or PSA levels (Table 3). To see whether AGR2 was an independent predictor of survival, we used a multivariate Cox model including dichotomized AGR2 expression, Gleason sum, PSA levels, and pT stage (Table 4). For high stage patients, AGR2 expression showed a trend toward significance (P = 0.087).

**Table 3:** Clinicopathologic parameters and AGR2 expression in patients with high stage prostate cancer

	Stage III/IV patients	Mean AGR2 expression (SE)	P-value	Low AGR2	High AGR2	P-value
Total cases	65	0.53 (0.057)		44	21	
Age at surgery			0.762^2^			0.763^1^
Median (range)	66 (50-76)			66 (50-76)	65 (50-73)	
Mean	64.6			64.8	64.3	
Gleason score			0.098^1^			0.161^3^
2-6	22	0.664 (0.104)		12	10	
7-10	43	0.457 (0.066)		32	11	
pT stage			0.451^1^			0.111^3^
pT2-pT3a	30	0.565 (0.087)		17	13	
pT3b	35	0.495 (0.076)		27	8	
Lymph node status			0.118^1^			0.480^3^
Positive	11	0.353 (0.118)		9	2	
Negative	54	0.563 (0.064)		35	19	
Tumor margins			0.990^1^			0.794^3^
Positive	35	0.536 (0.087)		23	12	
Negative	30	0.517 (0.072)		21	9	
Capsular invasion			0.805^1^			> 0.999^3^
No invasion	4	0.507 (0.174)		3	1	
Invasion	61	0.529 (0.060)		41	20	
PSA ng/mL			0.414^2^			0.089^1^
Median (range)	11.9 (2.1-96.5)		0.353^1^			0.092^3^
Mean	18.7					
< 10	25	0.607 (0.103)		14	11	
≥10	32	0.463 (0.073)		25	7	

^1^Mann-Whitney, ^2^Spearman Correlation, ^3^Fisher's Exact Test. Cases that were uninformative for a given variable were removed from the statistical analysis.

**Table 4:** Multivariate Cox model

	All Patients (n = 165)^2^			Stage III/IV Patients (n = 57)^3^		
Variable	HR	95% CI	P-Value	HR	95% CI	P-Value
AGR2 dichotomized	0.83	0.46 - 1.52	0.549	0.44	0.17 - 1.13	0.087
Gleason sum^1^	3.18	1.67 - 6.08	0.0005	1.14	0.42 - 3.07	0.795
Preoperative PSA	1.00	0.98 - 1.01	0.713	0.99	0.97 - 1.01	0.440
pT stage^2^	2.39	1.28 - 4.47	0.0064	2.57	1.00 - 6.65	0.051

^1^Gleason sum was dichotomized as low (< 7) vs. high (7-10).

^2 ^pT stage was dichotomized as pT2-pT3a vs. pT3b.

^3 ^In all patients, 165 of 187 cases were informative for all four variables.

^4 ^In stage III/IV patients, 57 of 65 cases were informative for all four variables.

## Discussion

AGR2 has been implicated in cancer pathogenesis and has been found to be up-regulated in multiple human cancers, including breast, lung, and prostate [13,14,18-20,30]. Our study has shown that AGR2 is higher in prostate cancer cells compared to non-malignant prostatic epithelial cells at the transcript and protein levels. This is consistent with other recent studies which have found an increase in AGR2 mRNA using microarray analysis of laser-capture microdissected cells and an increase in AGR2 protein levels [17-21,39,40]. Using TMA technology, we also verify a greatly enhanced AGR2 protein expression in malignant and early malignant PIN lesions compared to non-malignant epithelium. We observed that AGR2 levels were lower in advanced disease states as Gleason grade 5 spots expressed dramatically less AGR2 than grade 4 and grade 3 spots. Finally, we showed, for the first time, a novel predictive value for AGR2 expression with regard to tumor recurrence in individuals with higher stage disease. In this situation, relatively lower levels of AGR2 expression predicted a higher likelihood of tumor recurrence.

The exact biological role of AGR2 in humans is largely unknown. The AGR2 homolog in *X. laevis*, XAG2, has 73% similarity and 47% identity at the protein level [6] and plays a role in the differentiation of the mucus-secreting cement gland found on the anterior aspect of the frog embryo [41]. The newt homolog, nAG, has been shown to induce cellular proliferation in denervated limbs [42]. In the adult, AGR2 expression is restricted to a limited number of tissues and cell types in the body suggesting it is not a ubiquitously expressed product [43]. In the intestine, for example, AGR2 is found in the goblet cells and appears necessary for the production of intestinal mucus [44,45]. In malignant cells from organs such as the breast and prostate, AGR2 may be involved in additional functions. Interestingly, in various models of tumor progression, increased AGR2 promotes cell migration, invasion, and presumably metastatic spread [15,18,21,27,28,46,47]. However, the story may be more complicated as recently Bu, *et al*. observed that while over-expressed AGR2 in prostate cancer cells increased migration and invasion, it additionally repressed growth and proliferation [21]. The exact mechanistic role for AGR2 in these functions remains to be elucidated.

In our data, the lower expression of AGR2 in higher Gleason tumors appears to correlate with AGR2 being predictive of prostate cancer recurrence in individuals with higher stage cancers. Many studies report that AGR2 expression corresponds to a more differentiated phenotype [13,30,46]. Lower Gleason scores are indicative of well-differentiated tumors. If AGR2 indeed has opposing functions of promoting migration and invasion with higher expression yet allowing/promoting cellular proliferation upon decreased expression [21], it is intriguing to consider that our results reflect this balance of functional activity. We therefore hypothesize that in later stage prostate cancer, the balance towards increased proliferation (e.g., lower AGR2 expression) outweighs the need for enhanced migration and invasion (e.g., higher AGR2 expression). We are currently testing this hypothesis both *in vitro *and *in vivo*. Of interest Zhang *et al*., also used TMA technology to examine the associate of AGR2 expression levels with prostate cancer outcome [19]. Similar to us, they observed an increase in AGR2 expression in prostate cancer compared to normal or benign tissue. However, in apparent contrast to our results, they observed that increased AGR2 was associated with a poorer outcome [19]. While the explanation for this apparent discrepancy is unclear, it should be noted that Zhang *et al*. considered overall survival while our outcome measure was disease-specific recurrence. We did not have sufficient numbers of patients in our cohort who died from the disease in order to conduct meaningful statistical analysis of survival. Whether or not other subtle differences in treatment, clinical history, and/or demographics existed in the patient cohort at UCLA versus the Royal Liverpool University Hospital is difficult to assess. Nevertheless, whether the prognostic significance of AGR2 varies based on the outcome measurement (i.e., disease recurrence versus survival) warrants further investigation.

Our group, as well as others, is also examining AGR2 as a potential prostate cancer biomarker. In this regard, AGR2 has the following interesting attributes. First, it appears to be expressed at relatively high levels in individuals with prostate cancer; expression is lower from normal or non-malignant prostatic epithelium. Second, since AGR2 is secreted, there is the likelihood that the protein can be detected in blood or urine. Indeed Bu *et al*., have detected increased AGR2 transcript in urine sediments from prostate cancer patients [21]. That AGR2 is secreted is supported by our results as well as by mass spectrometry proteomic analysis of 2-D gel electrophoresis spots reported in the literature [48]. We are currently exploring whether AGR2 detected in body fluids is an accurate gauge for prostate cancer initiation, progression, and/or outcome. In particular, we are developing an assay to detect subnanogram per ml levels of AGR2. New monoclonal AGR2 antibodies need to be produced, however, since we found that the commercially available one did not recognize native AGR2 in solution.

## Conclusions

In summary, we found that AGR2 is a secreted protein expressed at relatively high levels by prostate cancer cells and cells in PIN lesions. We found that a relatively higher expression of AGR2 in patients with high stage prostate cancer stratified these individuals as less likely to have a tumor recurrence.

## Competing interests

The authors declare that they have no competing interests.

## Authors' contributions

ELM performed tissue immunoassays, statistical analysis and data interpretation, and manuscript preparation. VM provided statistical analysis and data interpretation. MA scored the TMA. LB performed the mRNA microarray analyses. SH provided oversight of statistical analyses. ESL performed Western blot analyses. LAK & YZ conducted software analysis, Western blot analyses, and ELISA. DC aided in study design. AYL & LG aided in study design, data interpretation, and manuscript preparation. All authors read and approved the final manuscript.

## Pre-publication history

The pre-publication history for this paper can be accessed here:

http://www.biomedcentral.com/1471-2407/10/680/prepub
